# Haber’s Syndrome: A Case Report

**DOI:** 10.7759/cureus.34906

**Published:** 2023-02-13

**Authors:** Sarah B Aljoudi, Mawaddah Tallab, Khalid Al Hawsawi

**Affiliations:** 1 Dermatology, King Abdulaziz University Faculty of Medicine, Jeddah, SAU; 2 Dermatology, King Fahad General Hospital, Jeddah, SAU; 3 Dermatology, King Abdulaziz Hospital, Makkah, SAU

**Keywords:** hyperpigmentation, photosensitivity, haber’s syndrome, dowling degos disease, reticulate pigmentary disorders

## Abstract

Haber's syndrome is an autosomal dominant, rare genodermatosis characterized by photosensitive, persistent facial erythema associated with reticulated hyperpigmentation. We present a case of an eight-year-old healthy Saudi girl who presented with facial erythema and generalized reticulated hyperpigmentation. Systematic review and laboratory studies were unremarkable. Histopathological examination revealed hyperpigmentation of the basilar keratinocytes with mild digitated elongations of the rete ridges. The patient was diagnosed with early-onset clinical presentation of Haber’s syndrome. In this report, Haber's syndrome is reviewed, and differential diagnoses of reticulated hyperpigmentation are discussed.

## Introduction

Reticulate pigmentary disorders are a group of genodermatoses inherited mostly in an autosomal dominant fashion [1]. They include the reticulate acropigmentation of Kitamura; Dowling-Degos disease and its variant, Galli-Galli disease; and Haber’s syndrome (HS).

HS is characterized by persistent, photosensitive, rosacea-like facial lesions manifesting in early adolescence [2], followed by the appearance of reticulate hyperpigmentation on the trunk, proximal extremities, and axillae. Other features include keratotic papules, comedo-like lesions, and pitted scars. There have been few reports of HS worldwide. Here we present a case of early-onset HS in a patient with rosacea-like facial eruptions and generalized reticulate hyperpigmentation.

## Case presentation

An eight-year-old female Saudi patient, otherwise healthy, visited the dermatology clinic with a four-year history of progressive, hyperpigmented skin lesions that started on the upper extremities and progressed to the trunk, face, and lower extremities. She reported a history of persistent, photosensitive facial eruption with no associated pain or burning sensation, noticed earlier than the hyperpigmented skin lesions. The patient had no history of recurrent infection or hospital admissions.

**Figure 1:**
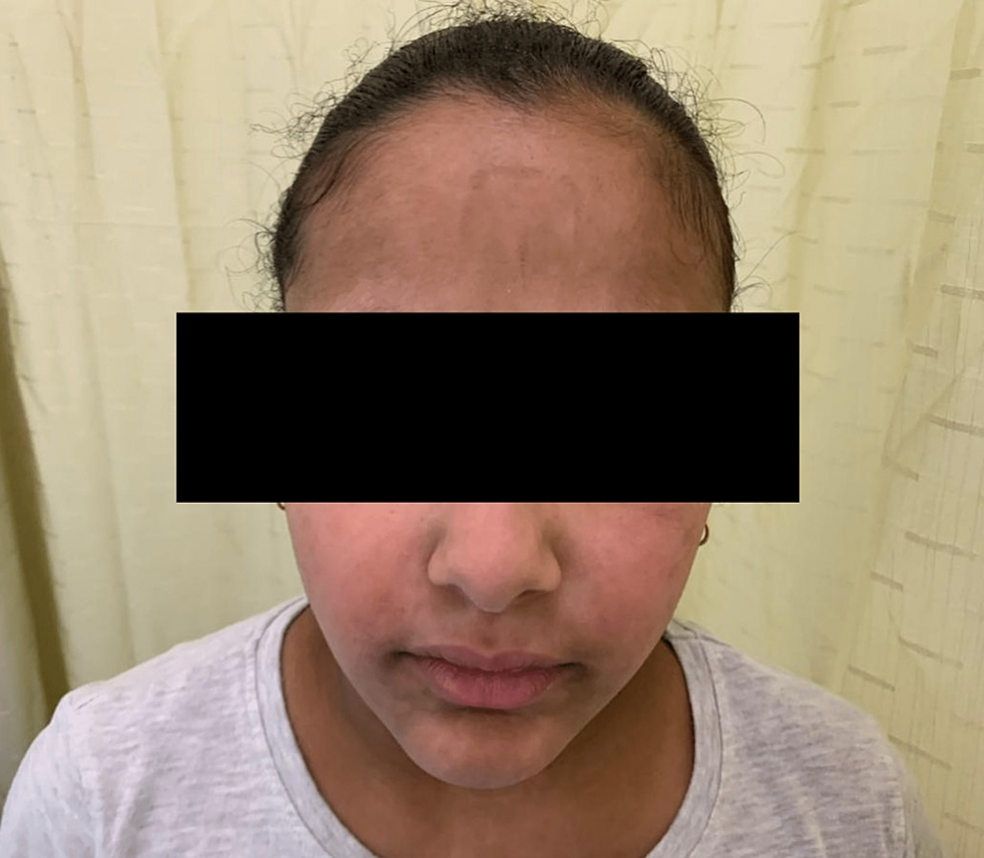
Haber’s syndrome. Rosacea-like facial eruption with telangiectasia in the studied case.

**Figure 2:**
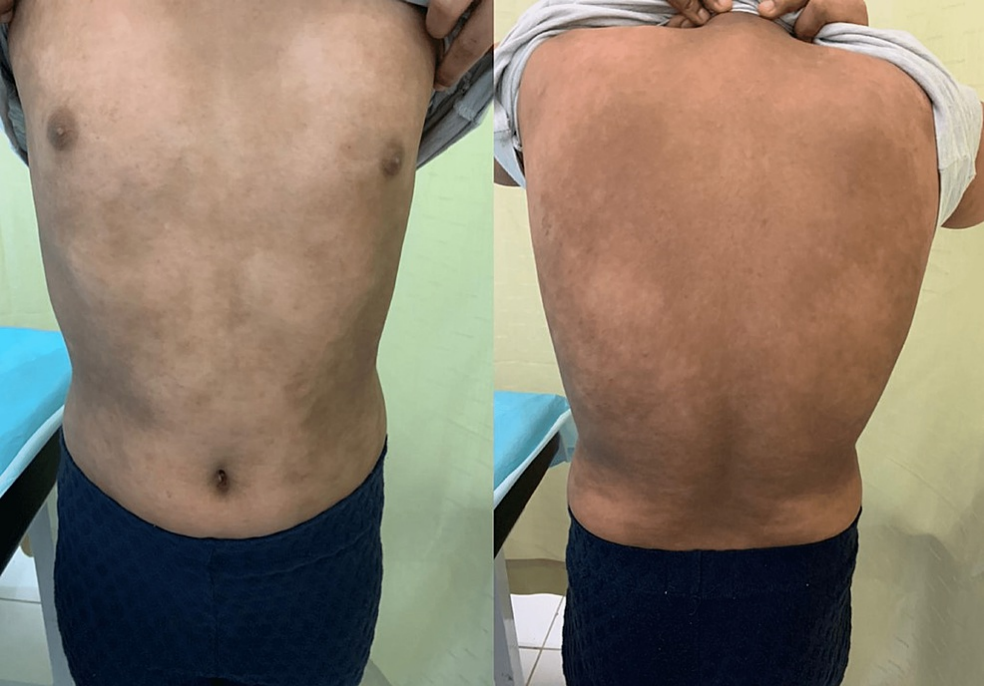
Haber’s syndrome. Reticulated hyperpigmentation of the trunk in the studied case.

The patient’s mother had similar rosacea-like skin lesions. The rest of her family was healthy, and all marriages on her paternal side were non-consanguineous. Dermatological examination revealed diffuse, hyperpigmented macules and patches in a reticulate pattern involving the face, extremities, and trunk, with prominent follicles under dermoscopy. Facial erythema, telangiectasia, and a few erythematous papules were observed on the patient’s cheeks, which is exacerbated by sun exposure (Figures 1, 2). No alopecia, comedones, or pitted scars were observed. Hair, nail, mucous-membrane, ocular, and dental examinations were all unremarkable. Her neonatal history was insignificant, her physical development was age-appropriate, and her academic performance was excellent.

**Figure 3:**
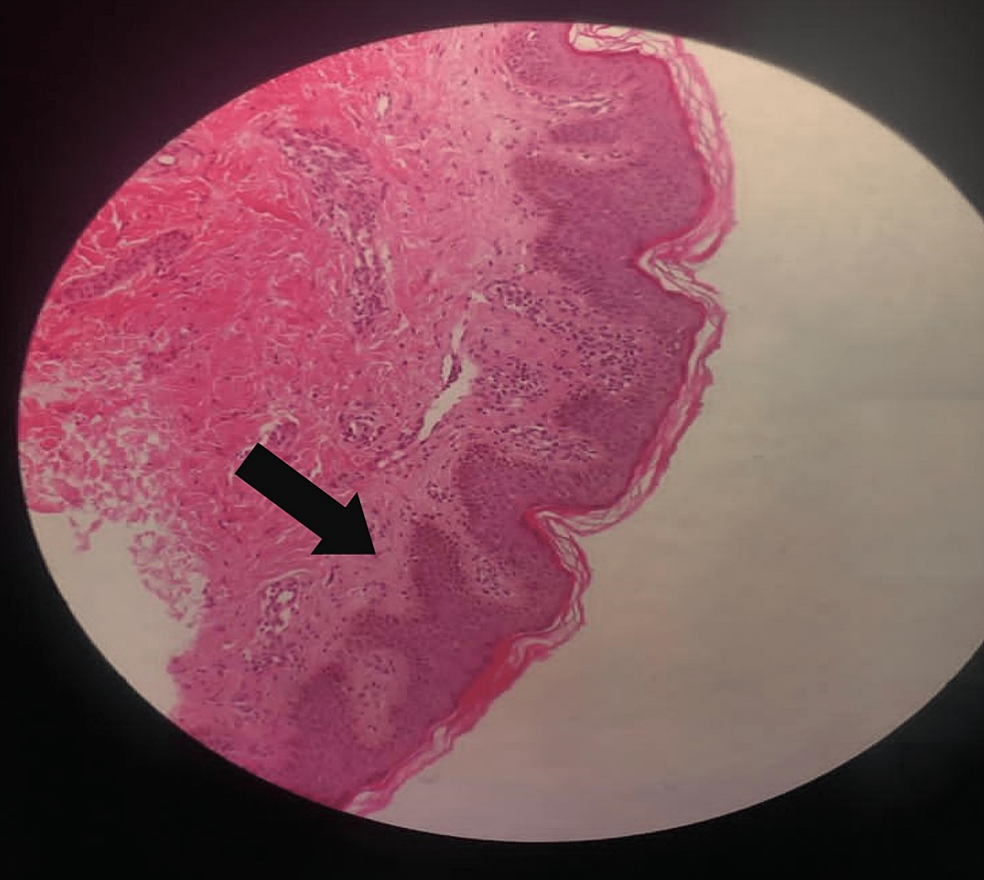
Haber’s syndrome. Histology of a macule in the right forearm revealing hyperpigmentation of the basal keratinocytes with mild digitate elongations of the rete ridges (arrow) (hematoxylin and eosin stain; magnification ×100)

Histopathological examination of a skin biopsy of the patient’s right lateral forearm revealed hyperpigmentation of the basal keratinocytes, with mild digitate elongations of the hyperpigmented rete ridges. A slight, patchy infiltration of lymphocytes was observed in the adjacent dermis (Figure 3). Amyloid staining results were negative.

Complete blood count, liver function, urea, creatinine, and electrolytes were all within normal limits. Genetic testing could not be performed at the facility due to limited resources, and the patient’s father declined such testing at another medical center. A diagnosis of HS was made, and the patient was counseled and advised to follow sun-protective measures. The patient was followed-up every six months and instructed to visit the clinic if new lesions or symptoms appeared. Written consent was taken from the patient and her guardian. 

## Discussion

HS was first described in 1965 as a rosacea-like eruption in three family members, and diagnostic criteria were suggested in 1988 [3]. It is an exceedingly rare genodermatosis. To our knowledge, only a few cases have been reported and published worldwide. The typical onset is during adolescence, when patients experience rosacea-like eruptions, potentially followed by hyperpigmented skin lesions later in life. However, the onset was much earlier in our patient, and the reticulate hyperpigmentation was generalized rather than localized to acral or flexural areas; this may be because of “genetic anticipation,” which occurs with autosomal dominant disorders, causing an earlier age and more severe phenotype than normal.

In reported cases [2-5], the clinical features of HS are inconsistent. All reported cases exhibited rosacea-like facial eruptions and familial concordance of similar skin lesions. Pigmentary skin lesions vary considerably among cases. Reported extra-facial features include keratotic lesions reminiscent of seborrheic keratosis, palmoplantar keratoderma, pitted scars, and comedones. In this case, histological examinations were of a supportive rather than diagnostic nature. However, the biopsy was obtained from an early lesion, which may explain the subtle changes observed in the histological examination.

The differential diagnosis in our case included diseases of generalized reticulate hyperpigmentation with onset in infancy and childhood, including dyskeratosis congenita, Naegeli-Franceschetti-Jadassohn syndrome, dermatopathia pigmentosa reticularis, and X-linked reticulate pigmentary disorder. It also included diseases with acral and flexural distributions, including Dowling-Degos disease and reticulate acropigmentation of Kitamura. However, these two types of diseases may appear together and are considered by certain authors to be different expressions of the same disorder [4]. The differential diagnosis of these reticulate pigmentary diseases is presented in Table 1.

**Table 1:** Differential diagnosis of Haber’s syndrome. AD: Autosomal dominant; DDD: Dowling–Degos disease; PPK: Palmoplantar keratoderma; SCC: Squamous cell carcinoma; XLR: X-linked recessive.

Disorder	Key features	Histopathology
Haber’s syndrome	Rosacea-like facial eruption plus the clinical features of Dowling-Degos disease.	Digitate elongations of the hyperpigmented rete ridges.
Dyskeratosis congenita	XLR (commonest form), the clinical triad of nail dystrophy, reticulated hyperpigmentation of the flexures, and oral leukoplakia. Other features: pancytopenia and increased risk of SCC.	Atrophy of the epidermis, mild interface vacuolization with melanophages in the upper dermis, and telangiectasia of the superficial vessels.
Naegeli–Franceschetti–Jadassohn syndrome	AD, fading reticulated hyperpigmentation, dental anomalies, PPK, hypohidrosis, and absent dermatoglyphics.	Clumps of melanin-laden melanophages are observed in the papillary dermis in a patchy distribution without overlying epidermal hyperpigmentation.
Dermatopathia pigmentosa reticularis	AD, persistent reticulated hyperpigmentation, PPK, alopecia, and hypoplastic dermatoglyphics.	
X-linked reticulate pigmentary disorder	XLR, early manifestations are neonatal colitis and recurrent pneumonia. Adults manifest generalized reticulated hyperpigmentation.	Amyloid deposits.
Dowling–Degos disease	AD, reticulated hyperpigmentation of flexural regions.	Digitate elongations of the hyperpigmented rete ridges. In DDD, thin, branching, heavily pigmented, downward proliferation also involves the infundibula of follicles and horn cysts.
Reticulate acropigmentation of Kitamura	AD, atrophic acral pigmentation, and palmoplantar pits.	

HS exhibits a considerable overlap of both histopathologic and clinical features with Dowling-Degos disease, which suggests that HS may be one of the facets of Dowling-Degos disease [3]. In Dowling-Degos disease, the pigmented macules and patches tend to coalesce and may involve the face, chest, and abdomen or be more extensive [6]. Associated features include pitted perioral scars, hyperpigmented comedones, hidradenitis suppurativa, multiple cysts and abscesses, and keratoacanthoma [7]. Neither HS nor Dowling-Degos disease exhibits substantial systemic involvement. However, certain authors consider the two to be clinically different entities [5-8]. This hypothesis is based on the fact that most of the reported cases of HS lack reticulate hyperpigmentation, and the hereditary onset of multiple seborrheic keratoses is not a common feature in reported cases of Dowling-Degos disease. However, the pigmentary changes observed in Dowling-Degos disease are caused primarily by mutations in keratin 5, which may be present in some cases of HS [8]. Additionally, the onset of multiple seborrheic keratoses during the first decade of life, as in our patient, is unusual.

Therapeutic options for HS include broad-spectrum sunscreen and other sun-protective measures, as well as the avoidance of triggers of facial erythema, such as smoking, alcohol ingestion, and excessive and prolonged application of corticosteroid ointment. Symptomatic treatment includes short-term use of topical steroids to alleviate any facial burning sensation [9,10]. Treatment of facial erythema includes classical therapies used to treat rosacea: the daily use of minocycline (100 mg) and metronidazole gel (0.75%) reportedly yields a satisfactory response [2]. Treatment of reticulate hyperpigmentation is often unsatisfactory: hydroquinone, tretinoin, and azelaic acid have been used with variable success. In cases exhibiting inflammation, topical corticosteroids, and calcineurin inhibitors may be used. Treatment is important to initiate to alleviate symptoms and enhance life quality. Genetic counseling can be offered to patients after genetic testing. 

## Conclusions

Haber’s syndrome typically manifests as a rosacea-like facial eruption at an early age and is exacerbated by sun exposure. A family history and histological features are further indications of this condition. Physicians should be aware of its manifestation and differential diagnosis, and research must continue to improve and find the most effective treatment of the disease beyond mere symptomatic treatment.
